# Amputations Resulting From Systemic Sclerosis: The Role of Multidisciplinary Cooperation

**DOI:** 10.7759/cureus.72280

**Published:** 2024-10-24

**Authors:** Rui Sousa, João Diniz, Inês Bernardo, Marlene Sousa, Mário Vaz

**Affiliations:** 1 Physical Medicine and Rehabilitation, Unidade Local de Saúde do Tâmega e Sousa, Penafiel, PRT; 2 Rheumatology, Unidade Local de Saúde do Tâmega e Sousa, Penafiel, PRT; 3 Physical Medicine and Rehabilitation, Unidade Local de Saúde de Santo António, Porto, PRT

**Keywords:** diffuse systemic sclerosis, major limb amputation, multidisciplinary management, prosthetic rehabilitation, vascular lesions

## Abstract

Systemic sclerosis (SSc) is a complex connective tissue disease involving microvasculopathy, immune dysregulation, and extensive organ fibrosis. It affects various systems including the skin, lungs, heart, and gastrointestinal tract. Management is challenging due to the disease's heterogeneity and often requires more than just pharmacological treatment. Severe cases may necessitate amputation due to complications like tissue necrosis.

This case report discusses the treatment of a 60-year-old man with cutaneous diffuse SSc who required multiple upper limb amputations due to progressive gangrene. Despite initial pharmacological and vascular interventions, the patient's condition led to metacarpophalangeal and transradial amputations. A multidisciplinary approach was crucial in determining the level of amputation and managing post-surgical rehabilitation. Following the transradial amputation, the patient participated in a comprehensive rehabilitation program to optimize functional recovery and prepare for prosthesis fitting.

The case highlights the need for a multidisciplinary strategy in managing severe SSc complications and underscores the importance of tailored rehabilitation in improving functional outcomes and quality of life for amputees.

## Introduction

Systemic sclerosis (SSc) is a connective tissue disease (CTD) characterized by vascular damage involving small blood vessels, reducing blood flow, and causing symptoms like Raynaud’s phenomenon and tissue damage. It also includes immune system activation with specific autoantibodies, such as anti-centromere and anti-Scl-70. Additionally, fibrosis, with excessive scarring in the skin and organs, leads to stiffening and organ failure over time. SSc is also divided into four forms: diffuse cutaneous, limited cutaneous, sine scleroderma, and overlap syndrome. SSc can affect the lungs, heart, kidneys, gastrointestinal (GI) tract, musculoskeletal system, blood vessels, and skin with digital ulcers and tissue loss [1,2]. The overall incidence rates globally range from 8 to 56 new cases per million persons per year, with prevalence rates falling between 38 and 341 cases per million persons [3]. This disease affects women more often, especially in middle age. Its exact cause is unknown but likely involves genetic and environmental factors [1].

Because of its complexity and heterogeneity, it is very challenging to treat SSc. Immunosuppressive therapy aims at the treatment of SSc-specific organ involvement, but it should be recognized that the field of management of patients with SSc is larger than pharmacological management alone [2].

In cases of severe involvement of microvasculature with the development of necrotic areas, amputation may be considered. The International Society for Prosthetics and Orthotics (ISPO) classification categorizes upper limb amputations based on the level or location, such as metacarpal, transmetacarpal, wrist disarticulation, transradial or below-elbow, elbow disarticulation, transhumeral or above-elbow, shoulder disarticulation, and forequarter amputation [4].

Considering the sudden alteration of physical integrity, disturbances in body image, and the impact on multiple aspects of the patient's life, it becomes essential to integrate the amputee into a rehabilitation program. The rehabilitation begins before prosthetization and concludes with the satisfactory use of the prosthesis, maximizing the functional potential. The rehabilitation program is a gradual process, requiring a multidisciplinary approach [5].

The authors describe a clinical case of a patient diagnosed with SSc who underwent multiple upper limb amputations and was integrated into a directed rehabilitation program.

## Case presentation

A 60-year-old man, right-handed, farmer, independent in daily activities with a history of active smoking, dyslipidemia, and arterial hypertension.

In February 2022, he was admitted to the rheumatology department for polyarthritis of the hands and feet, the Raynaud phenomenon, and four months of progressive skin thickening. Laboratory analysis revealed an antinuclear antibody title of 1/1280 and positives Scl70 and nor90, and capillaroscopy revealed a scleroderma-type pattern.

He was diagnosed with cutaneous diffuse SSc with joint, vascular, and skin involvement. The involvement of other organs was excluded. Pharmacological therapy with methotrexate weekly in progressive doses and nifedipine 30 mg daily and a multidisciplinary follow-up began with partial clinical improvement.

**Figure 1:**
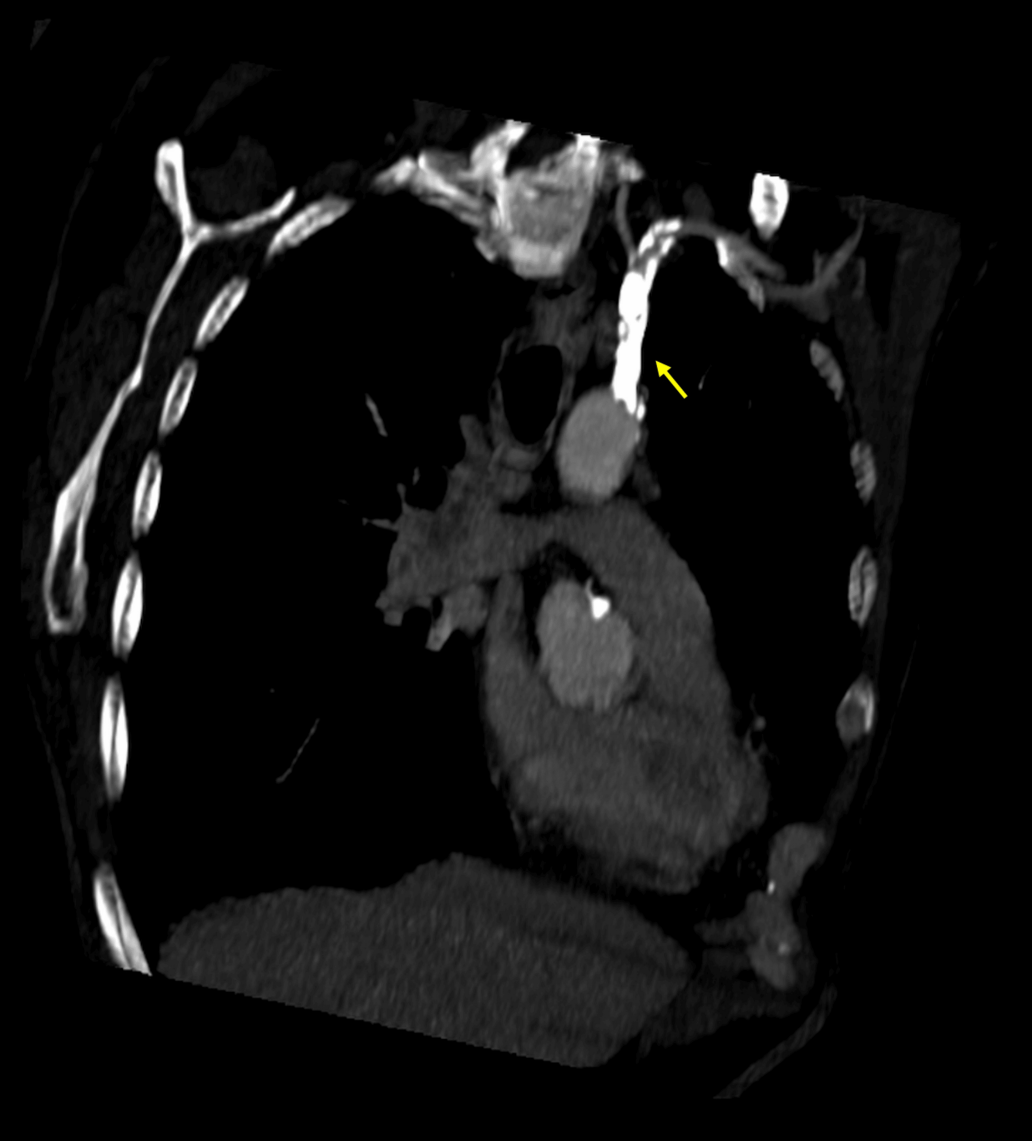
Coronal CT scan with the origin of brachiocephalic trunk and left subclavian artery with arteriosclerosis calcifications (yellow arrow)

**Figure 2:**
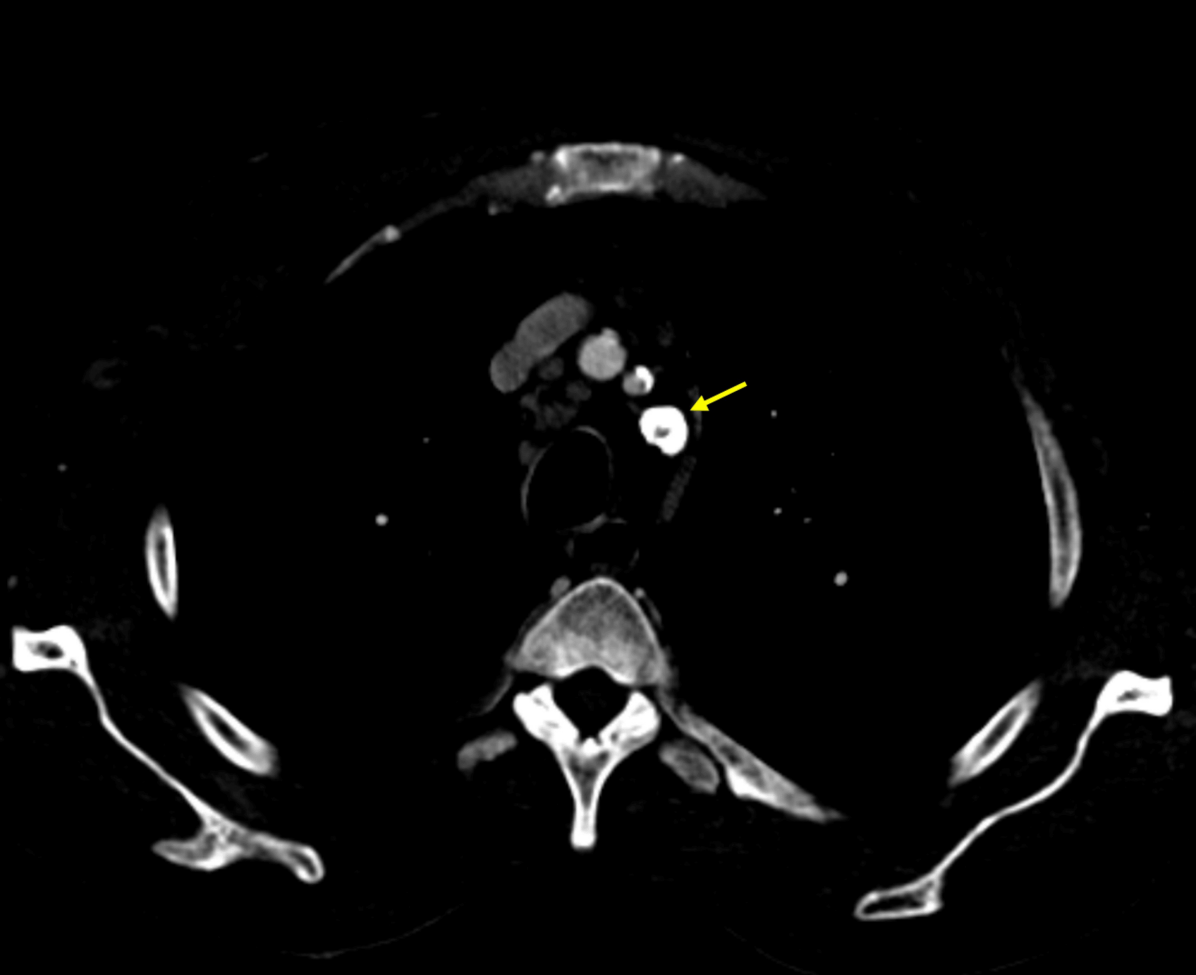
Axial CT scan of the left brachiocephalic trunk origin (yellow arrow)

In August 2022, in a vascular surgery consultation, he was diagnosed with asymptomatic left subclavian artery steal syndrome, and it was decided not to proceed with surgical treatment at that time. Figures 1-2 represent CT scans of this syndrome.

In November 2022, due to the appearance of a no-healing left second finger ulcer, he underwent an angiographic procedure with left subclavian stent placement. In the follow-up period, the stent showed patency, with a humeral pulse, no change in flow according to the Doppler ultrasound, and the angioCT confirming the patency of the stent.

In May 2023, for gangrene of the second, third, and fourth fingers of the left hand, he underwent metacarpophalangeal amputation of these fingers.

In September 2023, for gangrene of the fifth finger of the left hand, he underwent another metacarpophalangeal amputation.

In January 2024, due to necrosis of the left thumb and to decide the level of amputation surgery, preoperative evaluation by physical medicine and rehabilitation was requested. On examination, necrosis of the left thumb and signs of vascular insufficiency up to the left radiocarpal joint were noted. In terms of mobility, there was limited left wrist joint movement with 10° of extension and 10° of flexion, limited elbow extension with 30° flexion, passively irreducible, and submaximal pronosupination of the elbow. In terms of functionality, the patient exclusively used the left hand for stabilization and support and experienced limitations in instrumental tasks and daily activities such as dressing the upper body. Therefore, in a multidisciplinary meeting involving physical medicine and rehabilitation, orthopedics, and vascular surgery, it was decided to perform a transradial amputation of the left upper limb. An infundibuliform incision was made at the mid-third of the forearm, followed by consecutive sectioning of subcutaneous tissue and muscle fibers, isolation and ligature of the radial and ulnar arteries, identification and sectioning of underlying vascular, nervous, and tendinous structures, transverse osteotomy, and smoothing of bone edges.

Two months after the amputation surgery, the patient was evaluated in the physical medicine and rehabilitation - amputees clinic. Upon objective examination, the patient exhibited a deficit in active mobility at the bilateral glenohumeral joints, with an inability to perform overhead movements. Additionally, there was a reduction in active range of motion (ROM) at the knees, with the right knee demonstrating 110º of flexion and the left knee demonstrating 80º. The patient retained the ability to walk independently but experienced difficulties with transfers from the supine position to a seated position. At the time of the consultation, the patient showed partial dependence in the performance of activities of daily living (ADLs). Thus, the prosthetic fitting for the patient's lower limb was deferred, and the patient was enrolled in a comprehensive rehabilitation program aimed at restoring functionality and degree of autonomy.

**Figure 3:**
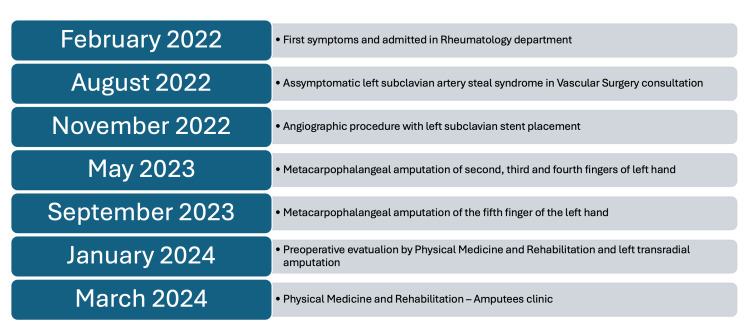
Timeline of key events

Figure 3 represents a timeline of the key events of this case presentation.

## Discussion

SSc is a chronic inflammatory, multi-organ disease. SSc is believed to result from environmental factors acting on a genetically predisposed individual, triggering a persistent, self-perpetuating process marked by vascular changes, inflammation, and autoimmunity, as well as fibrosis. The cell types most significantly involved in the disease process include endothelial cells, platelets, structural cells (such as pericytes, vascular smooth muscle cells, fibroblasts, and myofibroblasts), as well as immune cells (including T cells, B cells, monocytes, macrophages, and dendritic cells) [1].

The main causes of death associated with this condition are cardiorespiratory complications, but the involvement of small vessels can lead to necrosis of peripheral tissues, sometimes necessitating amputation surgery [6-8].

We presented a patient with SSc and multiple comorbidities who underwent transradial amputation surgery. The level of amputation had to be determined based on the functional evaluation of the most distal part of the limb as well as the state of the peripheral vasculature. In upper limb amputations, the length of the residual limb is crucial for defining the level of functionality and prosthetic adaptation. An amputation too proximal may compromise elbow pronosupination amplitudes, while one too distal may affect prosthetic adaptation [9]. Due to the presence of necrotic thumb lesions, signs of vascular insufficiency up to the radiocarpal joint, and functional deficit of the left wrist, the decision was made to perform transradial amputation at the junction of the mid-third with the distal third of the forearm.

After amputation surgery, it is crucial to promote tissue healing, pain control, and maintenance of joint ranges of motion. In the pre-prosthetic period, preparing the patient and the residual limb to receive the eventual prosthesis becomes important. This preparation includes multiple procedures such as stump molding, desensitization of the residual limb, maintaining joint ranges of motion, muscle strengthening, ADLs training, and exploring different prosthetic options [10-13].

Despite technological advances in the field of prosthetics, not all individuals undergoing amputation surgery are eligible for prosthesis placement [14]. Factors that may compromise prosthetic prescription include the presence of medical complications, functional deficit of the residual limb, unfavorable clinical prognosis considering the etiology, patient's preferences, and the existence of unrealistic expectations.

The primary goal of a prosthesis is to achieve body symmetry, and the fundamental requirements of prosthetic fitting, including comfort, functionality, and cosmesis, should be fulfilled.

## Conclusions

This case illustrates the profound impact of SSc on patient management, particularly when severe complications such as limb necrosis necessitate amputation. The multidisciplinary approach, combining pharmacological treatment, vascular interventions, and tailored surgical decisions, was pivotal in addressing the patient’s complex needs. Post-amputation, the comprehensive rehabilitation program played a crucial role in facilitating functional recovery and optimizing prosthetic adaptation. This case underscores the importance of integrating surgical, medical, and rehabilitative strategies to enhance the quality of life and functional outcomes in individuals with advanced SSc.
